# Analysis of influencing factors and construction of nomogram prediction model for pulmonary infection in patients with acute exacerbation of chronic obstructive pulmonary disease complicated with type II respiratory failure

**DOI:** 10.3389/fmed.2026.1842924

**Published:** 2026-06-29

**Authors:** Jianhuai Weng, Gaoyi Wu, Gaoze Zhang, Xiaohua Dai

**Affiliations:** Department of Emergency, Wenzhou Central Hospital, Wenzhou, China

**Keywords:** acute exacerbation of chronic obstructive pulmonary disease, influencing factors, nomogram, pulmonary infection, type II respiratory failure

## Abstract

**Objective:**

To construct a risk prediction nomogram model for pulmonary infection in patients with acute exacerbation of chronic obstructive pulmonary disease (AECOPD) complicated with type II respiratory failure.

**Methods:**

A retrospective analysis was conducted on clinical data of 361 patients with AECOPD complicated with type II respiratory failure. Restricted cubic spline analysis was used to analyze the nonlinear relationships between some continuous variables and the outcome. Single factor and multiple factor logistic regression analyses were used to determine independent influencing factors, the predictive model was constructed and the nomogram was drawn. ROC curve, calibration curve, and clinical decision curve were plotted to evaluate the predictive performance of the model.

**Results:**

Age showed no significant nonlinear relationship with concurrent pulmonary infection (P for nonlinearity > 0.05), whereas length of hospital stay, APACHE II score, and duration of combined antibiotic use all showed significant nonlinear relationships with concurrent pulmonary infection (P for nonlinearity < 0.05). Age, diabetes, hospital stay > 15 days, APACHE II score > 15 points, antibiotics combination time > 14 days were independent risk factors for pulmonary infection, and albumin was an independent protective factor (*P* < 0.05). In the training and validation cohorts, the AUCs of the ROC curves were 0.917 and 0.884, respectively. The predicted results of the nomogram model showed a certain consistency with the actual observed results. The Hosmer-Lemeshow goodness of fit test showed χ^2^ values of 2.858 and 5.162, with *P*-values of 0.943 and 0.740, respectively. The clinical decision curve results showed that the nomogram model exhibited a certain net benefit within the predictive risk threshold ranges of 0.05∼0.90 and 0.08∼0.79.

**Conclusion:**

The nomogram risk model constructed in this study may demonstrate a certain predictive performance in predicting pulmonary infection in patients with AECOPD complicated with type II respiratory failure, and may provide a reference for early clinical identification and intervention.

## 1 Introduction

Chronic obstructive pulmonary disease (COPD) is a common clinical condition characterized by emphysema and/or chronic bronchitis, caused by airflow limitation (1). It is currently the fourth leading cause of death worldwide and is projected to rise to the third by 2030, with patients experiencing acute exacerbation of COPD (AECOPD) facing a higher risk of mortality (2). AECOPD complicated by type II respiratory failure is a common clinical condition, occurring more frequently in the elderly population. Type II respiratory failure, also known as hypercapnic respiratory failure, is defined based on arterial blood gas analysis as resulting from inadequate alveolar ventilation leading to carbon dioxide retention and hypoxemia (3, 4). Patients with AECOPD complicated by respiratory failure often have numerous underlying comorbidities due to their condition. Additionally, treatment with corticosteroid medications can disrupt the patient’s immune-inflammatory balance, leading to abnormalities in lung fibrous structure, reduced pulmonary ventilation capacity, and an increased risk of pulmonary infection (5). The mortality rate after developing secondary pulmonary infection is high in patients with AECOPD complicated by respiratory failure, posing a serious threat to their lives (6). Therefore, investigating reliable and convenient prediction methods is crucial for controlling the risk of in-hospital mortality from secondary pulmonary infection in patients with AECOPD complicated by respiratory failure. Based on this, the present study aimed to identify risk factors for secondary pulmonary infection in patients with AECOPD complicated by type II respiratory failure by analyzing and screening relevant factors, construct a nomogram prediction model using these risk factors, and further evaluate the predictive performance of the model, with the goal of guiding timely and appropriate clinical interventions.

## 2 Patients and methods

### 2.1 Data source

**FIGURE 1:**
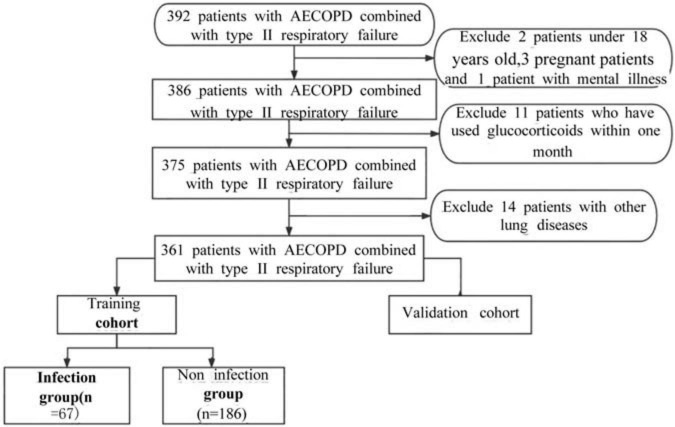
Patient collection process diagram.

This retrospective study included 361 patients with AECOPD complicated by type II respiratory failure admitted to our hospital between February 2023 and January 2025. Seventy percent of patients were assigned to the training cohort (*n* = 253), and 30% were assigned to the validation cohort (*n* = 108). Within the training cohort, patients were further divided into a non-infection group (*n* = 186) and an infection group (*n* = 67) according to the occurrence of new-onset pulmonary infection during hospitalization. A flowchart detailing patient selection is shown in Figure 1. This study was approved by the hospital’s Medical Ethics Committee. Missing data were assessed using Little’s MCAR test. The result showed P > 0.05, indicating that the missing data satisfied the MCAR assumption and were missing completely at random. Therefore, cases with missing data were removed using listwise deletion.

Inclusion criteria: ➀ Diagnosis of AECOPD confirmed according to the guidelines established by the Global Initiative for Chronic Obstructive Lung Disease (GOLD) (7); type II respiratory failure confirmed by arterial blood gas analysis, defined as PaO_2_ < 60 mmHg and PaCO_2_ > 50 mmHg; ➁ Patient age ≥ 18 years. Exclusion criteria: ➀ Pregnant or lactating patients; ➁ Patients with mental illness or other infectious diseases; ➂ Patients who used glucocorticoids within 1 month prior to the study; ➃ Patients with tuberculosis, lung tumors, non-infectious interstitial lung disease, pulmonary edema, atelectasis, pulmonary embolism, pulmonary vasculitis, or pulmonary eosinophilia; ➄ Patients who developed imaging findings combined with clinical symptoms within 48 h after admission were considered to have community-acquired infection or infection that had already existed before admission.

### 2.2 Clinical data collection

Clinical data were collected for all study subjects, including: gender, age, body mass index (BMI), duration of COPD, diabetes mellitus, hypertension, coronary heart disease, renal insufficiency, length of hospital stay (estimated length of hospitalization, cutoff value: 15 days (8), assessed within 24 h after admission), smoking history, alcohol consumption history, mechanical ventilation status, Acute Physiology and Chronic Health Evaluation II (APACHE II) score (cutoff value: 15 points (9)), Multiple Organ Dysfunction Syndrome (MODS) score, Pneumonia Severity Index (PSI) score, duration of combined antibiotic therapy (initial antibiotic strategy, cutoff: 14 days (10), assessed within 24 h after admission), platelet count (PLT), white blood cell count (WBC), C-reactive protein (CRP), albumin, arterial partial pressure of carbon dioxide (PaCO_2_), and arterial partial pressure of oxygen (PaO_2_). The APACHE II score consists of an acute physiology score, an age score, and a chronic health score, with a total possible score ranging from 0 to 71 points. Higher scores indicate greater disease severity, poorer prognosis, and higher mortality risk. The MODS scoring system includes functional assessment of six organ systems and is typically used to evaluate the severity of organ damage. The total score ranges from 0 to 24 points, with each organ system scored from 0 to 4 points. The PSI score is used to assess the severity of pneumonia and includes 20 parameters such as gender, age, and liver disease, with a total score ranging from 0 to 130 points. Higher PSI scores indicate more severe illness.

### 2.3 Assessment of pulmonary infection

Pulmonary infection was defined as newly developed hospital-acquired pneumonia occurring ≥ 48 h after hospitalization. The assessment process was as follows: (1) Baseline screening at admission (within 24 h after admission): Medical history collection, physical examination, routine blood tests, arterial blood gas analysis, and chest X-ray or CT examination were completed within 24 h after admission. If imaging infiltration was already present at admission and the patient met the clinical manifestations of infection, pulmonary infection was considered to have already existed at admission, and the patient was excluded. (2) Dynamic monitoring during hospitalization: During hospitalization, body temperature, sputum characteristics, white blood cell count, and respiratory symptoms were assessed daily. If any of the following suspected signs of infection occurred, chest imaging examination (X-ray or CT) was repeated: body temperature > 38.0°C or < 36.0°C; newly developed or aggravated purulent sputum; white blood cell count > 10 × 10^9^/L or < 4 × 10^9^/L; or worsening oxygenation that could not be explained by AECOPD itself, such as decreased PaO_2_ or further increased PaCO_2_. (3) Imaging diagnostic criteria: Chest CT or X-ray showing newly developed or progressive pulmonary infiltrates, meeting any of the following criteria: ➀ consolidation; ➁ ground-glass opacity; ➂ cavity formation or air bronchogram. 1.3.4 Microbiological criteria (auxiliary verification): Sputum culture showing pathogenic bacteria ≥ 10^5^ CFU/mL, with the same pathogen detected on repeated culture; positive blood culture; positive pleural effusion culture; or bronchoalveolar lavage fluid culture showing pathogenic bacteria ≥ 10^4^ CFU/mL. (4) Final determination and definition of onset time: Newly developed pulmonary infection during hospitalization was diagnosed when the hospitalization duration was ≥ 48 h, clinical manifestations of infection were present, and newly developed imaging infiltrates were observed. The onset time of infection was defined as the date when newly developed infiltrates were first confirmed by imaging.

### 2.4 Statistical analysis

Statistical analysis was performed using SPSS software (version 26.0; IBM Corp.) and R software (version 4.3.3). Continuous variables were expressed as mean ± standard deviation (mean ± SD) and compared between groups using the *t*-test. Categorical variables were expressed as counts and percentages [*n* (%)] and compared using the chi-square (χ^2^) test or Fisher’s exact test. Variables with *P* < 0.05 in the univariate analysis of the training cohort were included in the multivariate analysis. Some continuous variables showed nonlinear associations with the outcome in restricted cubic spline analysis; therefore, they were included in the regression model as binary variables. Multivariate logistic regression analysis was used to identify independent influencing factors. A nomogram was created using the “rms” package in R software. Internal validation was performed using the bootstrap method with 1,000 resamples. Receiver operating characteristic (ROC) curves were plotted to evaluate the model’s discrimination. Calibration curves were plotted to assess the model’s calibration. Clinical decision curve analysis (DCA) was used to evaluate the model’s clinical utility. A two-sided *P* < 0.05 was considered statistically significant.

## 3 Results

### 3.1 Comparison of training and validation cohort data

There were no statistically significant differences between the training cohort and the validation cohort in terms of gender, age, BMI, duration of COPD, diabetes mellitus, hypertension, coronary heart disease, renal insufficiency, length of hospital stay, smoking history, alcohol consumption history, mechanical ventilation status, APACHE II score, MODS score, PSI score, duration of combined antibiotic therapy, PLT, WBC, CRP, albumin, PaCO_2_, or PaO_2_ (all *P* > 0.05) (see Table 1).

**TABLE 1:** Comparison of training cohort and validation cohort data [*n* (%)/($\bar{x}$ ± s)].

Index	Training cohort (*n* = 253)	Validation cohort (*n* = 108)	χ^2^/*t*	*P*
Gender [*n*(%)]			0.494	0.482
Male	128(50.59)	59(54.63)		
Female	125(49.41)	49(45.37)		
Age (years)	62.87 ± 9.94	63.55 ± 10.02	0.594	0.553
BMI (kg/m^2^)	22.81 ± 3.10	22.94 ± 2.97	0.369	0.712
COPD course [*n*(%)]			0.871	0.647
<10 years	131(51.78)	59(54.63)		
10 years∼	82(32.41)	36(33.33)		
15 years∼	40(15.81)	13(12.04)		
Diabetes [*n*(%)]	30(11.86)	18(16.67)	1.518	0.218
Hypertension [*n*(%)]	80(31.62)	38(35.19)	0.437	0.509
Coronary heart disease [*n*(%)]	19(7.51)	6(5.56)	0.449	0.503
Renal insufficiency [*n*(%)]	12(4.74)	8(7.41)	1.027	0.311
Hospital stay [*n*(%)]			0.847	0.358
≤15 d	97(38.34)	47(43.52)		
>15 d	156(61.66)	61(56.48)		
Smoking [*n*(%)]	123(48.62)	51(47.22)	0.059	0.808
Drinking [*n*(%)]	25(9.88)	12(11.11)	0.124	0.724
Mechanical ventilation[*n*(%)]	109(43.08)	38(35.19)	1.956	0.162
APACHE II score [*n*(%)]			1.095	0.295
≤ 15 Points	123(48.62)	59(54.63)		
>15 Points	130(51.38)	49(45.37)		
MODS score [*n*(%)]			2.523	0.112
≤ 10 Points	163(64.43)	60(55.56)		
>10 Points	90(35.57)	48(44.44)		
PSI score [*n*(%)]			0.221	0.638
≤ 90 Points	155(61.26)	69(63.89)		
>90 Points	98(38.74)	39(36.11)		
Antibiotics combination time [*n*(%)]			0.757	0.384
≤14 d	153(60.47)	60(55.56)		
>14 d	100(39.53)	48(44.44)		
PLT ( × 10^9^/L)	108.10 ± 22.94	112.46 ± 23.05	1.651	0.100
WBC ( × 10^9^/L)	13.74 ± 2.36	13.39 ± 2.48	1.271	0.205
CRP (mg/L)	15.27 ± 4.91	14.68 ± 4.12	1.095	0.274
Albumin (g/L)	42.05 ± 6.10	41.77 ± 6.53	0.391	0.696
PaCO_2_ (mmHg)	59.29 ± 7.05	60.81 ± 7.47	1.842	0.066
PaO_2_ (mmHg)	52.34 ± 6.97	51.73 ± 7.06	0.758	0.449

### 3.2 Univariate analysis of concurrent pulmonary infection in AECOPD patients with type II respiratory failure in the training cohort

Within the training cohort, there were no statistically significant differences between the non-infection group and the infection group regarding gender, BMI, duration of COPD, hypertension, coronary heart disease, renal insufficiency, smoking history, alcohol consumption history, mechanical ventilation status, MODS score, PSI score, PLT, WBC, CRP, PaCO_2_, or PaO_2_ (all P > 0.05). The infection group had significantly higher age, a higher proportion of patients with diabetes mellitus, a higher proportion with length of hospital stay > 15 days, a higher proportion with APACHE II score > 15 points, and a higher proportion with duration of combined antibiotic therapy > 14 days compared to the non-infection group. Albumin levels were significantly lower in the infection group (all *P* < 0.05) (see Table 2).

**TABLE 2:** Univariate analysis of pulmonary infections in AECOPD patients with type II respiratory failure [*n* (%)/($\bar{x}$ ± s)].

Index	Non infection group (*n* = 186)	Infection group (*n* = 67)	χ^2^*/t*	*P*
Gender [*n*(%)]			0.099	0.753
Male	93(50.00)	35(52.24)		
Female	93(50.00)	32(47.76)		
Age (years)	60.15 ± 9.81	70.42 ± 10.23	7.264	0.000
BMI (kg/m^2^)	22.75 ± 3.10	22.98 ± 3.12	0.520	0.604
COPD course [*n*(%)]			0.567	0.753
<10 years	94(50.54)	37(55.22)		
10 years∼	61(32.80)	21(31.34)		
15 years∼	31(16.67)	9(13.43)		
Diabetes [*n*(%)]	12(6.45)	18(26.87)	19.640	0.000
Hypertension [*n*(%)]	62(33.33)	18(26.87)	0.953	0.329
Coronary heart disease [*n*(%)]	13(6.99)	6(8.96)	0.274	0.601
Renal insufficiency [*n*(%)]	8(4.30)	4(5.97)	0.047	0.829
Hospital stay [*n*(%)]			16.089	0.000
≤ 15 d	85(45.70)	12(17.91)		
>15 d	101(54.30)	55(82.09)		
Smoking [*n*(%)]	90(48.39)	33(49.25)	0.015	0.903
Drinking [*n*(%)]	16(8.60)	9(13.43)	1.291	0.256
Mechanical ventilation [*n*(%)]	74(39.78)	35(52.24)	3.115	0.078
APACHE II score [*n*(%)]			17.260	0.000
≤ 15 Points	105(56.45)	18(26.87)		
>15 Points	81(43.55)	49(73.13)		
MODS score [*n*(%)]			1.537	0.215
≤ 10 Points	124(66.67)	39(58.21)		
>10 Points	62(33.33)	28(41.79)		
PSI score [*n*(%)]			3.129	0.077
≤ 90 Points	120(64.52)	35(52.24)		
>90 Points	66(35.48)	32(47.76)		
Antibiotics combination time [*n*(%)]			20.452	0.000
≤14 d	128(68.82)	25(37.31)		
>14 d	58(31.18)	42(62.69)		
PLT (× 10^9^/L)	107.81 ± 23.62	108.90 ± 22.45	0.328	0.743
WBC (× 10^9^/L)	13.60 ± 2.46	14.12 ± 2.33	1.504	0.134
CRP (mg/L)	14.92 ± 4.61	16.19 ± 6.02	1.776	0.077
Albumin (g/L)	43.95 ± 6.36	36.78 ± 5.41	8.216	0.000
PaCO_2_ (mmHg)	58.92 ± 6.94	60.31 ± 7.28	1.387	0.167
PaO_2_ (mmHg)	52.85 ± 7.12	50.94 ± 6.78	1.906	0.058

### 3.3 Dose-response relationships between selected variables and concurrent pulmonary infection

**FIGURE 2:**
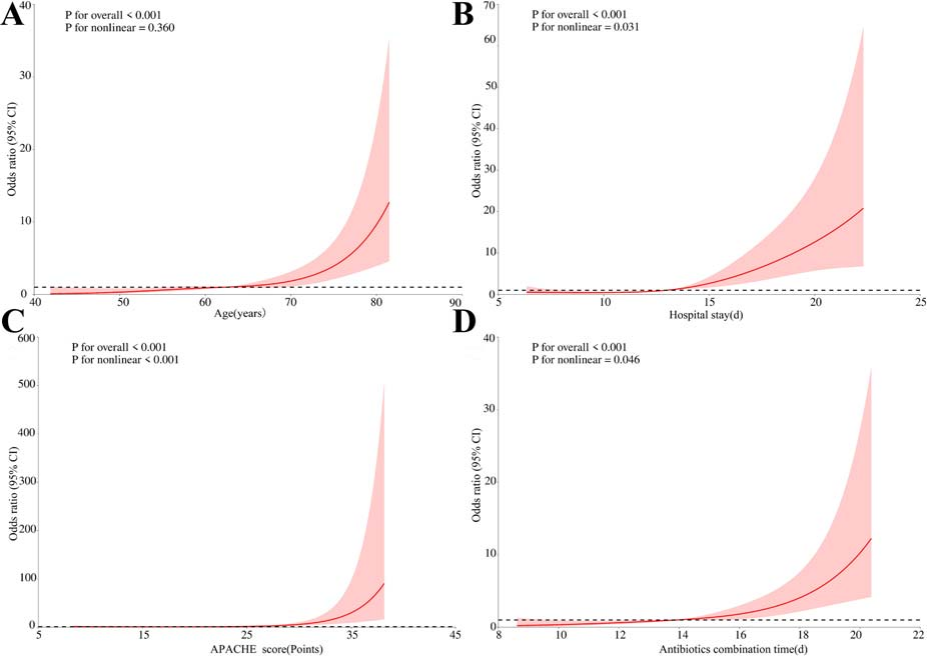
Restricted cubic spline plots showing the associations between selected continuous variables and concurrent pulmonary infection in AECOPD patients with type II respiratory failure. **(A)** Age; **(B)** length of hospital stay; **(C)** APACHE II score; **(D)** duration of combined antibiotic therapy.

The results of restricted cubic spline analysis showed that age (Figure 2A) had no significant nonlinear relationship with concurrent pulmonary infection (P for nonlinearity > 0.05), whereas length of hospital stay (Figure 2B), APACHE II score (Figure 2C), and duration of combined antibiotic use (Figure 2D) all had significant nonlinear relationships with concurrent pulmonary infection (P for nonlinearity < 0.05).

### 3.4 Logistic regression analysis of concurrent pulmonary infection in AECOPD patients with type II respiratory failure

To avoid overfitting, only variables found to be statistically significant in Table 2—namely age (continuous variable, entered as original value), diabetes mellitus (Yes = 1, No = 0), length of hospital stay (> 15 days = 1, ≤15 days = 0), APACHE II score (>15 points = 1, ≤15 points = 0), duration of combined antibiotic therapy (>14 days = 1, ≤14 days = 0), and albumin (continuous variable, entered as original value)—were included as independent variables in the multivariate logistic regression model. The VIF values of all independent variables were <10, indicating no multicollinearity. The occurrence of secondary pulmonary infection in patients with AECOPD complicated by type II respiratory failure was the dependent variable (Infection = 1, No infection = 0). The results showed that age, diabetes mellitus, length of hospital stay > 15 days, APACHE II score > 15 points, and duration of combined antibiotic therapy > 14 days were independent risk factors for pulmonary infection, while albumin level was an independent protective factor (all *P* < 0.05) (see Table 3).

**TABLE 3:** Logistic regression analysis screening for independent factors of pulmonary infection in AECOPD patients with type II respiratory failure.

Influence factor	β	*SE*	Wald χ^2^	OR	95%CI	*P*	VIF
Age	0.105	0.021	25.310	1.110	1.066∼1.157	0.000	2.149
Diabetes	1.179	0.558	4.457	3.250	1.088∼9.706	0.035	1.868
Hospital stay	1.162	0.481	5.838	3.195	1.245∼8.198	0.016	2.075
APACHE II score	1.484	0.431	11.842	4.410	1.894∼10.266	0.001	2.894
Antibiotics combination time	1.365	0.411	11.051	3.917	1.751∼8.761	0.001	1.639
Albumin	−0.225	0.045	24.894	0.798	0.731∼0.872	0.000	2.574
Constant	−11.371	11.986	7.476	0.000	∼	0.000	–

### 3.5 Construction of the nomogram for concurrent pulmonary infection in AECOPD patients with type II respiratory failure

**FIGURE 3:**
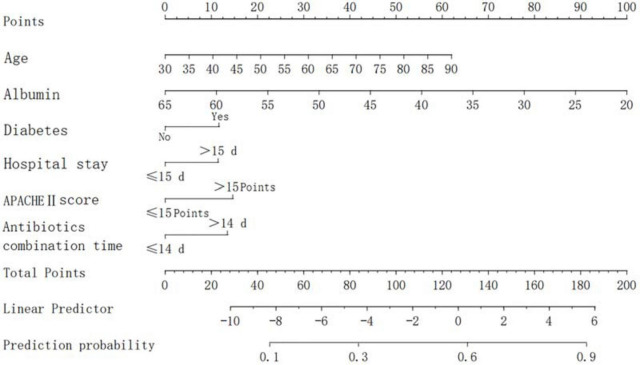
Nomogram for predicting pulmonary infection in AECOPD patients with type II respiratory failure. Locate the specific numerical value or category of the patient on each axis of the predictor and draw a vertical line upward to the “Points” axis at the top, reading the corresponding single-item score. Sum all single-item scores to obtain the “Total Points.” Draw a vertical line leftward from the “Total Points” axis to the “Prediction probability” axis at the bottom, reading the final probability of infection occurrence.

Based on the independent risk factors identified in Table 3, a prediction model was established. The model formula is: logit(P) = −11.371 + (0.105 × Age) + (1.179 × Diabetes Mellitus) + (1.162 × Length of Hospital Stay > 15 days) + (1.484 × APACHE II Score > 15 points) + (1.365 × Duration of Combined Antibiotic Therapy > 14 days) − (0.225 × Albumin). Based on the weights assigned to different factors derived from the training cohort, a nomogram was generated using R software to predict the risk of secondary pulmonary infection in patients with AECOPD complicated by type II respiratory failure (see Figure 3).

### 3.6 Validation of the nomogram prediction model in the training and validation cohorts

**FIGURE 4:**
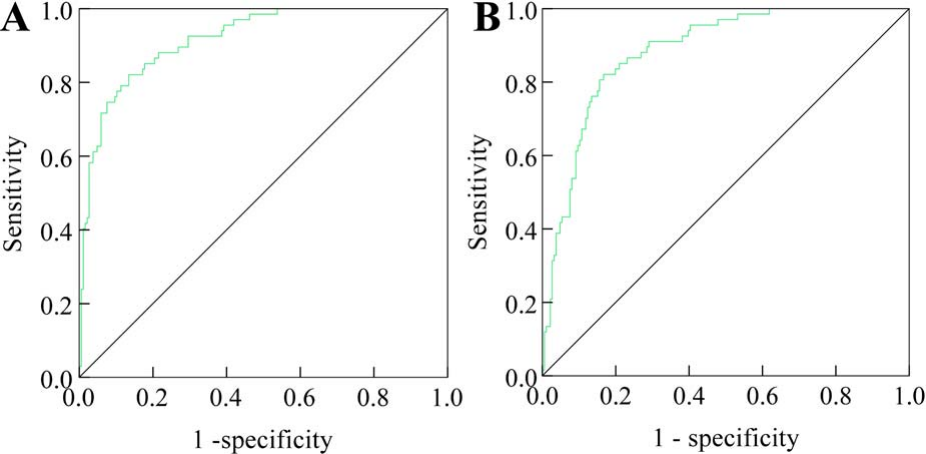
ROC curve of the nomogram for predicting pulmonary infection in AECOPD patients with type II respiratory failure (**A:** training cohort, **B:** validation cohort).

**FIGURE 5:**
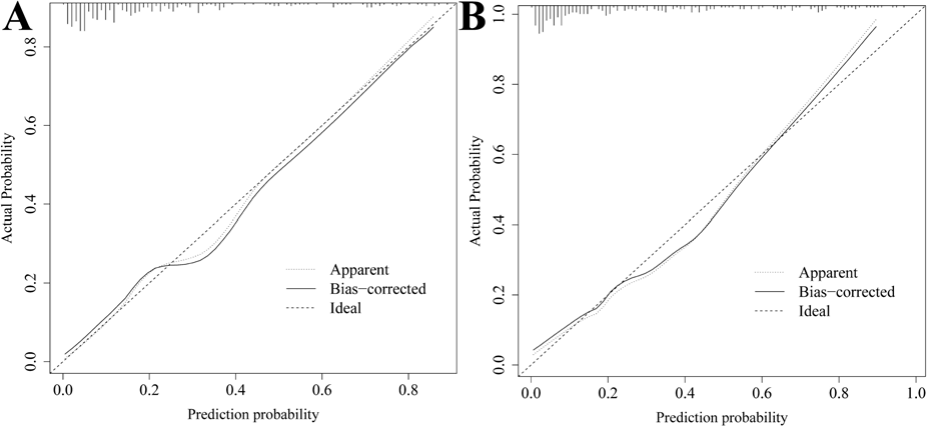
Calibration curve of the nomogram for predicting pulmonary infection in AECOPD patients with type II respiratory failure (**A:** training cohort, **B:** validation cohort).

**FIGURE 6:**
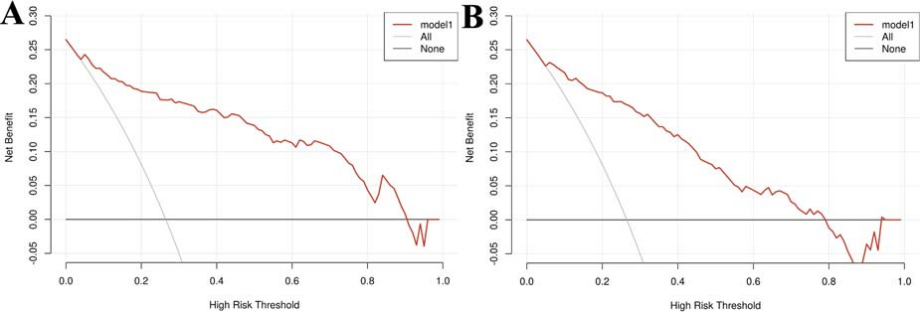
Clinical decision curve of the nomogram for predicting pulmonary infection in AECOPD patients with type II respiratory failure (**A:** training cohort, **B:** validation cohort).

Internal validation was performed using the Bootstrap method with 1,000 resamples. ROC curve analysis showed that the Area Under the Curve (AUC) was 0.917 (95% CI: 0.881–0.954) in the training cohort and 0.884 (95% CI: 0.842–0.927) in the validation cohort, At the optimal cutoff values of 0.42 and 0.40 in the training and validation cohorts, respectively, the sensitivity was 82.46 and 82.37%, the specificity was 85.71 and 81.05%, the positive predictive value was 78.24 and 77.69%, and the negative predictive value was 88.09 and 87.62%, respectively, indicating good model discrimination (see Figure 4). Calibration curve validation demonstrated high consistency between the nomogram-predicted probabilities and the actual observed outcomes in both the training and validation cohorts. The Hosmer-Lemeshow goodness-of-fit test yielded χ^2^ = 2.858 (*P* = 0.943) for the training cohort and χ^2^ = 5.162 (P = 0.740) for the validation cohort. The calibration slopes were 0.99 and 0.98, the calibration intercepts were 0.01 and 0.02, and the Brier scores were 0.13 and 0.11 in the training and validation cohorts, respectively (see Figure 5). Decision curve analysis (DCA) results showed that the nomogram demonstrated good net benefit in both the training and validation cohorts across a wide range of threshold probabilities, specifically within the ranges of 0.05–0.90 and 0.08–0.79, respectively. When the clinical decision threshold was set at 0.3, that is, intensified anti-infective therapy was initiated when the predicted probability of infection was ≥ 30%, the net benefit was significantly improved (see Figure 6).

## 4 Discussion

Pulmonary infection is one of the common disease complications in respiratory and critical care departments, and it readily affects the respiratory system and other organs (11). Although treatments related to pulmonary infection have made considerable progress over the past decade, severe pulmonary infections can trigger multiple organ dysfunction, shock, and even threaten life in patients (12). Therefore, pulmonary infection remains a major cause of infection and mortality globally, with its mortality rate increasing from approximately 20% to over 50% (13). This study, by collecting clinical data from 361 patients with AECOPD complicated by type II respiratory failure, found that age, diabetes mellitus, length of hospital stay, APACHE II score, duration of combined antibiotic therapy, and albumin level may be independent influencing factors for concurrent pulmonary infection in these patients.

In this study, older age was identified as a risk factor for concurrent pulmonary infection, consistent with the findings of Sundh et al. (14), whose study found that risk factors for COVID-19 infection in patients with chronic respiratory failure included higher age, cardiovascular disease, and kidney disease. With increasing age, the body’s immune function tends to decline, and the function of various organs also decreases, increasing the probability of pathogen invasion into the respiratory tract and possibly increasing the risk of pulmonary infection (15). (2) The results from Huang et al. (8) showed that age ≥ 60 years, length of hospital stay ≥ 15 days, diabetes mellitus, and use of ≥2 antimicrobial drugs are risk factors for pulmonary infection in COPD patients. Similarly, this study found that diabetes mellitus (OR = 3.250) and length of hospital stay (OR = 3.195) were predictors in the nomogram model. Diabetes mellitus can reduce the body’s immunity and its ability to clear pulmonary pathogens, cause microvascular lesions in lung tissue, and thereby may increase the risk of pulmonary infection (16). Furthermore, patients with AECOPD have inherently damaged airways and reduced ability to defend against exogenous pathogens. Coupled with the abundance of pathogens in the hospital environment, prolonged hospital stays increase the risk of pulmonary infection. (3) The APACHE II score not only reflects the patient’s physiological function but can also indicate their nutritional status. A higher APACHE II score signifies greater bodily consumption, poorer immune function, higher disease severity, and consequently, a possibly greater risk of pulmonary infection (9, 17). (4) Research by Han et al. (10) similarly showed that hypoalbuminemia, duration of antibiotic use ≥ 14 days, and blood glucose ≥ 11.10 mmol/L upon admission are risk factors for secondary lower respiratory tract fungal infection in AECOPD patients. In our study, duration of combined antibiotic therapy > 14 days also influenced pulmonary infection. This is primarily because prolonged antibiotic use can disrupt the normal microbial balance within the body, leading to dysbiosis, increased antimicrobial resistance among some pathogens, and consequently, a possible increase in the probability of pulmonary infection. Therefore, in clinical practice, patients should strictly adhere to medication prescriptions. If combination therapy is necessary, clinicians must rigorously be aware of contraindications for drug combinations to avoid adverse outcomes. (5) A study by Dilixiati et al. (18) confirmed that albumin < 40 g/L, fever, and asthma are important influencing factors for concurrent community-acquired pneumonia in AECOPD patients. Our results also indicate that albumin is a protective factor against concurrent pulmonary infection and may be an important predictor in the model (OR = 0.798). Serum albumin is an objective indicator of the body’s nutritional status and is closely related to immunity. When serum albumin levels decrease, immunity declines, nutritional status worsens, the lung tissue’s ability to resist pathogen invasion diminishes, and the risk of pulmonary infection may increases. Previous studies have reported (19, 20) that supranormal LVEF may reflect underlying pathological conditions, such as systemic inflammation, and may be associated with poorer clinical outcomes. However, LVEF was not included in the univariate analysis or in the predictive model in the present study. Future studies with larger sample sizes are needed to further comprehensively evaluate the predictive value of this indicator. In addition, the single-center study design may introduce certain bias, and the results of the multivariate analysis still need to be validated by multicenter studies with larger sample sizes.

Wang et al. (21) constructed a nomogram risk prediction model for bacterial infection in AECOPD patients, achieving AUCs of 0.835 and 0.785 in the training and validation sets, respectively. A nomogram model constructed in a study by Liu et al. (22) demonstrated good discrimination and accuracy in predicting prognosis and respiratory failure in patients with severe pulmonary infection, with an AUC of 0.813 in their validation. Validation of the nomogram model constructed in our study revealed AUCs of 0.917 and 0.884 in the training and validation cohorts, respectively. The relatively high AUCs may also be related to model overfitting and require further validation in future studies. Furthermore, our nomogram showed a certain degree of consistency between predicted results and actual observations, along with a certain net benefit; however, these results were validated using data from the same center, and external validation is still needed. This confirms that the nomogram model developed in our study provides clinicians with a potential tool and may play a certain role in assessing the risk of concurrent pulmonary infection in patients with AECOPD complicated by type II respiratory failure. Its usage is simple and convenient, visually reflecting the risk of disease occurrence, which facilitates effective communication between doctors and patients’ families. Its evaluation performance needs to be further validated in future studies with larger sample sizes.

This study has some limitations. (1) It was a single-center study. Although internal validation was performed using the Bootstrap method, external validation was not conducted. In the future, we will collaborate with other centers to externally validate the model. (2) Some data were missing. Pulmonary function data were not included in this study; however, various scores reflecting disease severity and arterial blood gas analysis indicators were included, which partially compensates for the impact of missing pulmonary function indicators on the model. (3) As a retrospective analysis, it is susceptible to selection bias and missing data. (4) In this study, due to the lack of procalcitonin (PCT) data, PCT was not included as a predictive variable. However, PCT is an important diagnostic marker for bacterial infection, and its absence from the model might have reduced the model’s predictive performance. (5) The sample size of this study was relatively limited, and the relatively high AUC may be related to a potential risk of overfitting. Therefore, further validation in studies with larger sample sizes is warranted. Future prospective studies will aim to address these limitations and further refine the nomogram prediction model.

In summary, a nomogram prediction model was developed based on routine laboratory tests and clinical record data and, after validation with larger-sample data in future studies, may be useful for the early prediction of the risk of concurrent pulmonary infection in patients with AECOPD complicated by type II respiratory failure. Its six risk variables include age, diabetes mellitus, length of hospital stay, APACHE II score, duration of combined antibiotic therapy, and albumin. This model may have certain value in assisting clinical medical and nursing staff in implementing targeted treatment and nursing measures and improving clinical outcomes.

## Data Availability

The original contributions presented in this study are included in this article/supplementary material, further inquiries can be directed to the corresponding author.
